# Unifying frequency metrology across microwave, optical, and free-electron domains

**DOI:** 10.1038/s41467-025-62808-5

**Published:** 2025-09-24

**Authors:** Yujia Yang, Paolo Cattaneo, Arslan S. Raja, Bruce Weaver, Rui Ning Wang, Alexey Sapozhnik, Fabrizio Carbone, Thomas LaGrange, Tobias J. Kippenberg

**Affiliations:** 1 Institute of Physics, Swiss Federal Institute of Technology Lausanne (EPFL), Lausanne, Switzerland; 2 Center for Quantum Science and Engineering, Swiss Federal Institute of Technology Lausanne (EPFL), Lausanne, Switzerland; 3 Institute of Electrical and Micro-Engineering, Swiss Federal Institute of Technology Lausanne (EPFL), Lausanne, Switzerland

**Keywords:** Frequency combs, Transmission electron microscopy, Integrated optics, Applied physics

## Abstract

Frequency metrology lies at the heart of precision measurement. Optical frequency combs provide a coherent link uniting the microwave and optical domains in the electromagnetic spectrum, with profound implications in timekeeping, sensing and spectroscopy, fundamental physics tests, exoplanet searches, and light detection and ranging. Here, we extend this frequency link to free electrons by coherent modulation of the electron phase by a continuous-wave laser locked to a fully stabilized optical frequency comb. Microwave frequency standards are transferred to the optical domain via the frequency comb, and are further imprinted in the electron spectrum by optically modulating the electron phase with a photonic chip-based microresonator. As a proof-of-concept demonstration, we apply this frequency link in the calibration of an electron spectrometer and verify its precision by measuring the absolute optical frequency. This approach achieves a 20-fold improvement in the accuracy of electron spectroscopy, relevant for investigating low-energy excitations in quantum materials, two-dimensional materials, nanophotonics, and quantum optics. Our work bridges frequency domains differed by a factor of  ~ 10^13^ and carried by different physical objects, establishes a spectroscopic connection between electromagnetic waves and free-electron matter waves, and has direct ramifications in ultrahigh-precision electron spectroscopy.

## Abstract (web summary)

Frequency metrology lies at the heart of precision measurement. Here, authors establish a phasecoherent frequency link across microwave, optical, and free-electron domains. This bridges electromagnetic waves and electron matter waves, advancing ultrahigh-precision electron spectroscopy.

## Introduction

Frequency is the most accurately measured physical quantity and is pivotal to precision metrology in a multitude of scientific and technological sectors. It was advised by Arthur Schawlow, the Nobel laureate in physics in 1981, to “never measure anything but frequency”^1^. The optical frequency comb (OFC) consists of a “comb” of precisely equidistant frequency lines at optical frequencies separated by a microwave frequency^2,3^. The OFC serves as a phase-coherent link, or a “light gear”, that accurately connects frequency components in the microwave and optical realms, and has revolutionized a plethora of fields, including atomic clocks^4,5^, spectroscopy^6–8^, ultrafast optics^9^, telecommunications^10^, fundamental physics tests^11,12^, exoplanet searches^13–15^, navigation and ranging^16–18^.

Electron spectroscopy uses free electrons to perform spectroscopic measurements of specimens. In particular, electron energy-loss spectroscopy (EELS) measures the energy loss of electrons transmitting through and inelastically scattered by a specimen, based on the spatial distribution of the electron beam bent and dispersed by a magnetic field^19,20^. Typically implemented in a transmission electron microscope (TEM) with a post-column spectrometer, EELS possesses sub-atomic spatial resolution and is a powerful probe for local excitations or chemically resolved imaging, widely applied in the investigation of atomic composition^21^, chemical bonding^22,23^, and vibrational properties^24,25^ of materials, macromolecule assemblies and subcellular compartments in biological systems^26^, as well as thermal^27^, electronic^28^, and optical^29^ properties of nanoscale devices. Despite the excellent spatial resolution, the spectral resolution of EELS (about 10^−3^ eV for state-of-the-art techniques^30^) is far inferior to that of its optical counterparts, where the optical frequency comb allows measuring absolute frequencies with precision reaching mHz-level or 10^−18 ^eV^31^. The limited spectral resolution significantly hinders precise and quantitative characterization of fine structures in EELS spectra, making it challenging to extract crucial chemical information, such as oxidation states and local bonding environments. Additionally, this reduced resolution compromises the analysis of low-energy collective dynamics, including phonons, excitons, and optical properties. These factors are essential for advancing research and development in quantum materials, two-dimensional materials, nanoelectronics, and nanophotonics. In spite of advances in monochromators and aberration correctors for improving the spectral resolution, the energy reference or calibration standard for EELS still largely relies on elemental edges associated with inner shell ionizations^19,32^. This type of calibration has a limited accuracy susceptible to chemical shift of the edge position, which can differ by several eV in different publications^33^, and needs to presume a constant dispersion that overlooks local irregularity and nonlinearity from electron dispersion and instrumental limitations. Alternatively, EELS can be calibrated by the voltage applied to the drift tube of the electron spectrometer^34–38^. However, this method is vulnerable to the instabilities of the drift tube voltage and the primary electron energy, and the drift tube itself requires additional calibration.

**Fig. 1:**
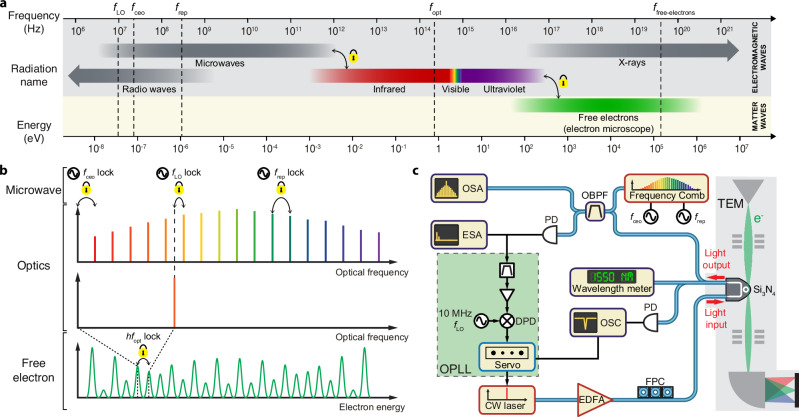
Frequency metrology across microwave, optical, and free-electron domains. **a** Frequency range of electromagnetic waves and free-electron matter waves. **b** Frequency locking scheme across microwave, optical, and free-electron domains. Microwave and optical domains are connected by a fully stabilized optical frequency comb with the carrier-envelope-offset frequency *f*_ceo_ and the repetition rate *f*_rep_ being stabilized. A continuous-wave (CW) laser is offset locked to one comb tooth via a local oscillator (LO) at frequency *f*_LO_. The CW laser then coherently modulates the phase of an electron beam, generating sidebands in the electron spectrum that are regularly spaced by the photon energy *hf*_opt_. **c** Experimental setup. A CW laser pumps a Si_3_N_4_ photonic chip-based microresonator in a transmission electron microscope (TEM) to modulate the electron phase and broaden the electron spectrum measured in a post-column electron spectrometer. A wavelength meter performs coarse measurement of the laser frequency. The transmitted CW laser is mixed with a stabilized optical frequency comb (OFC) to generate microwave beatnotes that are measured with an electronic spectrum analyzer (ESA). An optical spectrum analyzer (OSA) monitors the laser and the OFC. An optical phase-locked loop (OPLL) offset-locks the laser to one comb tooth. The beatnote and a 10 MHz microwave local oscillator (LO) are mixed at a 12-bit double-balanced digital phase detector (DPD) that accounts for large phase slips induced by frequency fluctuations. The DPD output, or error signal, is fed to a servo controller to adjust the laser frequency. Blue paths represent fiber connections, and black lines stand for electronic connections. EDFA erbium-doped fiber amplifier, FPC fiber polarization controller, PD photodetector, OSC oscilloscope, OBPF optical bandpass filter.

Optical frequency references are ubiquitous in precision measurement. We establish a frequency link across microwave, optical, and free-electron realms, bridging electromagnetic and matter waves and connecting frequency scales differed by a factor of  ~10^13^ (Fig. 1a). By doing so, we unite electron spectroscopy with optical frequency metrology and demonstrate EELS calibration with precisely measured photon energy, which offers unprecedented accuracy and the ability to resolve nonlinearity and irregularity.

## Results

### Frequency metrology across microwave, optical, and free-electron domains

Figure 1b illustrates the concept of frequency metrology uniting microwave, optical, and free-electron domains. The microwave and optical frequencies are linked by an optical frequency comb, whose carrier-envelope-offset frequency *f*_ceo_ and repetition rate *f*_rep_ are stabilized. Therefore, the comb teeth are located at optical frequencies that are precisely synthesized from microwave frequencies: *f*_m_ = *f*_ceo_ + *m* × *f*_rep_, with *m* the mode number. A monochromatic continuous-wave (CW) laser is then offset-locked to one comb tooth with a microwave local oscillator (LO), and one obtains a synthesized optical frequency

1 $${f}_{{{{\rm{opt}}}}}={f}_{{{{\rm{ceo}}}}}+m\times {f}_{{{{\rm{rep}}}}}\pm {f}_{{{{\rm{LO}}}}}.$$

The CW laser is then used to impose a coherent phase modulation onto free-electron wavefunctions^39–41^. The electron-light coupling leads to a sinusoidal phase modulation of the electron wavefunction, which broadens the initially narrow electron spectrum into a comb-like structure consisting of regularly spaced energy sidebands (see SI) at

2 $${E}_{{{{\rm{N}}}}}={E}_{0}+N\times h{f}_{{{{\rm{opt}}}}},$$

where *E*_0_ is the initial electron energy, *h* is the Planck constant, and *N* is an integer. Equally, the generation of energy sidebands can be understood as quantum-mechanical superpositions of electron states corresponding to the absorption or emission of *N* photons in the inelastic electron-light scattering (IELS) process. In the end, one obtains an electron spectrum consisting of energy sidebands centered at the initial electron energy *E*_0_ with energy change

3 $${E}_{{{{\rm{N}}}}}-{E}_{0}=Nh({f}_{{{{\rm{ceo}}}}}+m{f}_{{{{\rm{rep}}}}}\pm {f}_{{{{\rm{LO}}}}}),$$

arising from electron phase modulation at an optical frequency synthesized from microwave frequencies.

We use a high-quality-factor Si_3_N_4_ photonic chip-based microresonator^42,43^ driven by a CW laser to implement the electron phase modulation (Fig. 1c). The combination of electron microscopy and integrated photonics has recently enabled continuous-beam electron phase modulation^44^, cavity-mediated electron-photon pairs^45^, and electron probing of nonlinear dissipative structures^46^. Though coherent optical modulation of free electrons was achieved more than a decade ago in the context of photon-induced near-field electron microscopy^39^, it is the development of photonic integrated circuit-based electron phase modulation^44^ that permits both CW laser-driven operation and strong phase modulation via resonance enhancement and electron-light phase matching. These attributes translate into a narrow-linewidth driving laser with a well-defined frequency *f*_opt_ and a large electron spectral broadening, both important to the application of precise optical frequency metrology in electron spectroscopy.

**Fig. 2:**
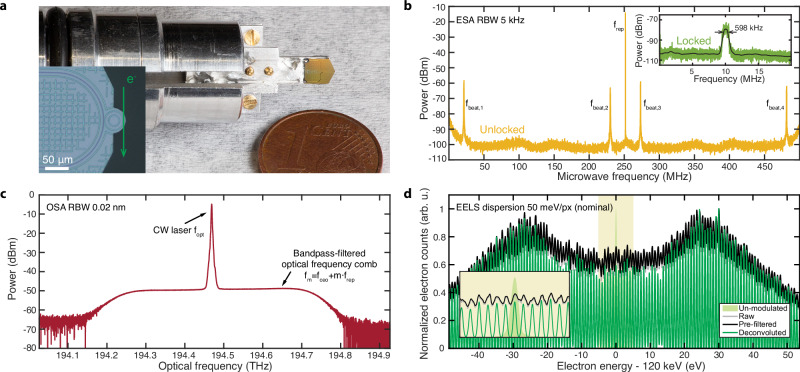
Frequency-locked microwave, optical, and electron spectra. **a** Photograph of the photonic chip mounted on a custom holder. Inset: optical micrograph of the microresonator and schematic depiction of the electron beam position. **b** Microwave spectrum recorded with an ESA. The beatnote around 250 MHz is from the repetition rate *f*_rep_ of the optical frequency comb, and the other beatnotes are generated by beating the CW laser with the frequency comb teeth. Inset: microwave spectrum when the OPLL is activated; the smoothed beatnote shows a 3-dB bandwidth of 598 kHz. **c** Optical spectrum recorded with an OSA. The narrow peak corresponds to the CW laser and the lower plateau is from the bandpass-filtered frequency comb. Individual comb lines are not resolved by the OSA. **d** Electron spectrum recorded with the electron spectrometer. The raw spectrum (gray) is first smoothed (black) with a Gaussian pre-filter, and then deconvolved to generate a comb-like spectrum with well-separated peaks (green). The electron spectrum without optical modulation (light green shaded area) is illustrated for comparison. Inset: zoomed-in view of the central part of the spectra.

The experimental setup (Fig. 1c) consists of a TEM with a post-column electron energy-loss spectrometer, a photonic chip-based microresonator mounted on a custom holder (Fig. 2a), and an optical frequency synthesizer based on a frequency comb. Efficient interaction requires the electron velocity to match the phase velocity of light (phase-matching condition). In the present case, electrons (~120 keV energy, corresponding to  ~2.9 × 10^19^ Hz frequency) phase-matched to the optical wave (~1550 nm wavelength) traverse the evanescent near-field of the microresonator and are spectrally broadened by the IELS^44^. An optical phase-locked loop (OPLL) locks the CW laser to one tooth of a stabilized OFC with a 10 MHz offset frequency defined by a microwave LO, so that the absolute laser frequency is precisely determined. Figure 2b shows a typical microwave spectrum with multiple beatnotes. The beatnote near 250 MHz originates from the repetition rate *f*_rep_ of the OFC, while the other 4 beatnotes are generated by beating the CW laser with the comb teeth of the OFC. The absolute optical frequency can be precisely measured from the microwave beatnote and the mode number *m* determined by the wavelength meter. When the OPLL is activated, the beatnote with the lowest frequency is locked to the LO at *f*_LO_ = 10 MHz, and the microwave spectrum is displayed in the inset of Fig. 2b. In this way, an absolute optical frequency is synthesized. Figure 2c illustrates the corresponding optical spectrum. Note that OSA only monitors the optical frequency components, while precise frequency measurement is performed in the microwave domain. The optical spectrum features a narrow peak from the CW laser, and a plateau from the bandpass-filtered OFC (individual comb lines cannot be resolved).

Figure 2d demonstrates an example of the measured electron spectrum generated by the IELS. Due to the finite spectral width (0.7–0.8 eV) of the incident electrons and hence the elastically scattered electrons (known as the zero-loss peak, ZLP), the energy sidebands at multiples of the photon energy (~0.8 eV) are not fully separated and overlap each other. To mitigate this issue, we first smooth the raw data by convolution with a Gaussian pre-filter, and then use the Richardson-Lucy deconvolution^47^ to retrieve a comb-like spectrum with well-separated peaks. By separating the electron energy sidebands and eliminating the influence of a slightly asymmetric ZLP shape, the deconvolution enhances the precision of sideband position localization, serving as accurate energy markers generated by optically modulated free electrons. These electron spectral peaks are spaced by a photon energy *hf*_opt_, with the absolute optical frequency *f*_opt_ precisely measured or synthesized from microwave frequencies *f*_rep_, *f*_ceo_, and *f*_LO_ (cf. Eq. (3)). Consequently, a frequency link is built across microwave, optical, and free-electron domains.

### Ultrahigh-precision calibration of an electron spectrometer

As a proof-of-concept demonstration, we apply the aforementioned frequency metrology to the calibration of an EELS spectrometer attached to an un-monochromated TEM with a thermionic electron gun. Recent advances in electron monochromators, spectrometers, and detectors have pushed the energy resolution of EELS to the millielectronvolt level^30^. However, the typical calibration methods of electron spectrometers fall short of the required precision or demand complicated operations. The standard calibration based on ionization edges^19,32^ has a limited spectral range and accuracy and is insensitive to local changes of dispersion. The drift tube scanning method^34–38^ could perform local calibration, but suffers from multiple acquisitions and voltage instabilities.

The frequency metrology across microwave, optical, and free-electron domains provides a direct frequency link from precisely measured microwave frequencies to the change of the electron energy (cf. Eq. (3)). Therefore, the IELS-broadened electron spectrum serves as an energy reference for EELS calibration, using the energy sidebands located at multiples of the photon energy *hf*_opt_ as calibration markers.

**Fig. 3:**
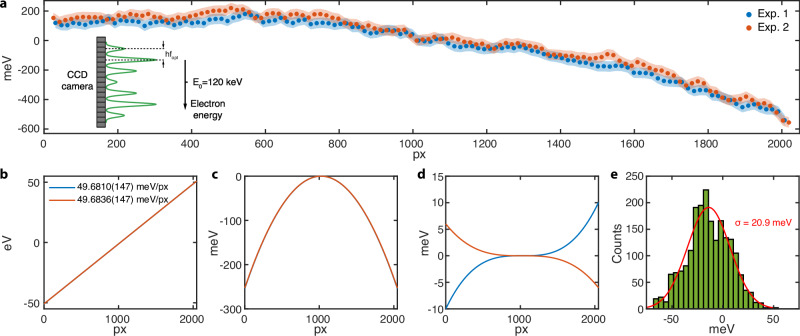
Electron spectrometer calibration with two independent exposures. **a** Deviations of the calibration markers at multiples of the photon energy from the nominal energy of the spectrometer for Exposures (Exp.) 1 and 2. Shaded areas correspond to error bars. Inset: a schematic depiction of obtaining the energy solution from the electron spectrum. **b**–**d** First, second, and third-order components of the polynomial fits of the energy solutions (pixel-to-energy mappings) for the two exposures. The first-order components illustrate the calibrated average dispersion. **e** Histogram of the pixel-wise root-mean-square deviation between the energy solutions of the two exposures. Red curve represents a normal distribution fit with a standard deviation of 20.9 meV.

We employ the IELS-broadened electron spectrum in calibrating an electron spectrometer with 2048 pixels set at 50 meV/px nominal dispersion (Fig. 3). The goal of the calibration is to obtain an “energy solution”, a precise pixel-to-energy mapping that might differ from the nominal dispersion specified by the manufacturer. Pixel positions (with sub-pixel precision) for the calibration markers are assigned an energy of the corresponding multiples of the photon energy; pixels in between the markers are assigned an energy from linear interpolation. Figure 3 shows the calibrations with two independent exposures, both using electrons modulated by a CW laser at a frequency of 194 567 516 MHz. Figure 3a displays the deviation of the calibration markers (at multiples of the photon energy) from the nominal energy that is linear with the pixel number with a slope of 50 meV/px. Both calibrations reveal a negative deviation indicating the nominal dispersion overestimates the true dispersion and that the common assumption of having a constant dispersion is inaccurate. Figure 3b–d depict the first, second, and third-order components of the polynomial fits of the energy solutions for the two exposures. The first-order components represent the calibrated average dispersion, which is  ~300 μeV/px systematically less than the nominal dispersion, and differs by less than 3 μeV/px between the two calibrations with a time interval of 4 min 40 s. The deviation from the nominal energy solution is dominated by a second-order component common in both exposures, while the third-order component is at the scale of data scattering. Figure 3e shows a histogram of the pixel-wise root-mean-square deviation between the energy solutions retrieved from the two exposures, and a normal distribution fit (red curve) indicates a standard deviation of 20.9 meV. The difference in the calibrated energy dispersions and the standard deviation of the energy solutions are indicative of the “jitter” between two measurements. The similarity between the two exposures in local irregularities (Fig. 3a, e), calibrated dispersion (Fig. 3b), and global nonlinearity (Fig. 3c) cross-verifies the accuracy and precision of our frequency-metrology-based calibration method.

**Fig. 4:**
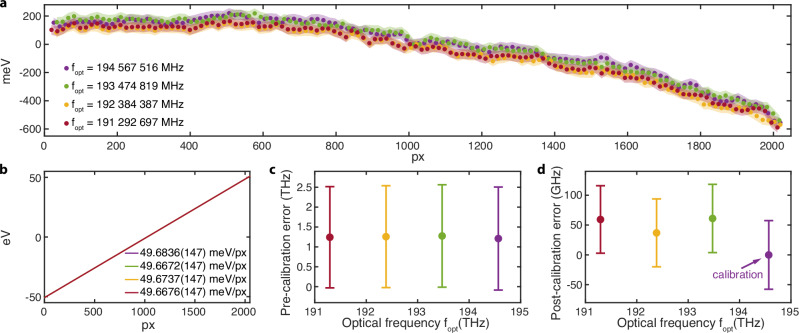
Electron spectrometer calibrations with electrons modulated at four different optical frequencies. **a** Deviations of the calibration markers at multiples of the photon energy from the nominal energy of the spectrometer. Shaded areas correspond to error bars. **b** First-order components of the polynomial fits of the energy solutions (pixel-to-energy mappings) for the four calibrations, showing the calibrated average dispersions. Errors in optical frequencies measured from electron spectra before (**c**) and after (**d**) calibrating the electron spectrometer with electrons modulated at the optical frequency of 194 567 516 MHz. Error bars in (**c**) are derived from the maximal deviation of the pre-calibration nominal dispersion from the calibrated dispersion, and error bars in (**d**) are obtained from the calibration uncertainty of the average dispersion.

We further compared the spectrometer calibrations using electrons modulated at four different absolute optical frequencies: 194 567 516 MHz, 193 474 819 MHz, 192 384 387 MHz, and 191 292 697 MHz (with measurement time intervals of 25 min 25 s, 18 min 17 s, and 15 min 19 s). Figure 4a illustrates the deviation of the calibration markers from the nominal energy for the four calibrations. The calibrated average dispersions, or the first-order components of the polynomial fits of the energy solutions, are shown in Fig. 4b. Similarly to the previous case, the calibrated average dispersions reveal a systematic error of  ~300 μeV/px in the nominal dispersion, and differ from each other by less than  ~20 μeV/px.

We verify the accuracy of electron-based frequency metrology by measuring the absolute optical frequency from the IELS-broadened electron spectrum. This measurement also marks the first achievement of referencing electron spectroscopy to a precise frequency standard. With the number of electron energy sidebands and their energy span, one could back-calculate the photon energy and thus the optical frequency. Figure 4c shows the error in such measurement for the four optical frequencies, using the nominal or pre-calibrated pixel-to-energy mapping of the electron spectrometer. The errors are in the range of 1.20–1.28 THz, corresponding to a fractional error of about 6–7 parts in 10^3^. Alternatively, we calibrate the spectrometer using electrons modulated at the optical frequency of 194 567 516 MHz, and the resultant measurement errors in optical frequency become less than 61.1 GHz, corresponding to a fractional error less than 3.2 parts in 10^4^. We emphasize that this value does not represent the precision limit of such measurement, as it does not exclude the effects of instrument performance, instability, and lab environment. We also point out that the frequency-resolving capability in our demonstration is directly provided by free electrons, in stark contrast to previous reports where the frequency-resolving capability of high-precision electron spectroscopy comes from tuning the laser frequency^44,48^.

## Discussion

We unify frequency metrology across microwave, optical, and free-electron domains. The cascaded frequency link is enabled by an optical frequency comb that unites microwave and optical frequencies and a photonic chip-based electron phase modulator that bridges optical waves and free electrons. Our work connects frequency domains differed by a factor of  ~10^13^ and carried by different physical objects - electromagnetic waves and free-electron matter waves. As a proof-of-concept demonstration, we apply this frequency link to the calibration of an electron spectrometer. The standard calibration technique utilizes core-loss excitations from reference samples as the energy reference. This method is widely adopted, as outlined in textbooks and the international standard (ISO-24639) for EELS calibration procedures. Despite its simplicity, this technique is affected by shifts in core-loss energies that depend on the specific chemical environment of the sample, known as the chemical shift^23,33,49^. This energy uncertainty propagates into the EELS calibration, resulting in an energy solution uncertainty of  ~1 eV. Moreover, this standard method relies on only two energy markers and assumes a constant dispersion across the spectrometer, rendering it incapable of detecting nonlinearities and local irregularities (Table 1). An alternative calibration approach employs an energy reference derived from the voltage applied to the drift tube of the spectrometer^34–38^. This method involves incrementing the drift tube voltage in discrete steps and recording a spectrum for each voltage level, thereby retrieving the energy solution without assuming a constant dispersion. However, this approach necessitates multiple acquisitions, making it vulnerable to instabilities in the drift tube voltage, variations in the primary beam energy, and short-term drifts caused by the instrument and laboratory environment. Furthermore, the drift tube itself requires calibration, and any associated uncertainties propagate into the EELS calibration (Table 1).

**Table 1:** Comparison of our method with other calibration techniques for EELS spectrometers

Energy reference	Optical frequency	Core-loss edge	Drift tube voltage
References	Our work	Refs. ^19,32^	Refs. ^34–38^
Energy solution uncertainty	35.08 meV	≳1 eV	≳100 meV
Dispersion uncertainty	14.68 μeV/px	≳1 meV/px	≳100 μeV/px
Number of acquisitions	Single	Single	Multiple
Measure irregularities and nonlinearities?	Yes	No	Yes
Verification by a precise energy standard?	Yes	No	No

Notably, only in our study are the calibration results verified by a precise energy standard with an uncertainty below 600 kHz (2.48 neV). The calibration uncertainties of other methods are estimated from the uncertainties associated with their respective energy references.

The calibration technology presented in our work offers several key advantages (Table 1). We employ an optical frequency synthesizer to generate the energy reference with extreme accuracy. This optical frequency remains unaffected by sample composition, instrument drift, or laboratory conditions, and it does not require additional calibration. The photonic microresonator-based electron phase modulator provides an efficient electron-photon coupling so that a low-power CW laser generates energy sidebands covering the full range of the spectrometer and a single acquisition is sufficient for calibrating the whole spectrometer, making the approach robust against voltage instabilities and short-term drift. Having over a hundred energy markers in a single spectrum also enables the measurement of spectrometer nonlinearities and local irregularities. Furthermore, the performance of the calibration is independently verified through precise measurements of other optical frequencies. This verification protocol is agnostic to the specific calibration method and sets a benchmark for the careful evaluation and comparison of various techniques. Our calibration method is conceptually analogous to the use of optical frequency combs in astronomical spectrometer calibration^13,14^ (“astrocombs”), a landmark technology that has revolutionized astrophysics by precision optical frequency metrology. Just as astrocombs provide a precise frequency ruler for calibrating optical spectrometers, our approach employs the energies of the IELS photon sidebands as precise energy markers for calibrating electron spectrometers, introducing optical frequency metrology to electron spectroscopy.

We demonstrate the technology using an un-monochromated TEM equipped with a thermionic electron gun operating in the continuous-beam mode, which indicates that the technique could be applied in almost any existing TEM and EELS spectrometers, boosting tremendously the resolution of affordable instruments and having a major, broad impact on both public funded research and characterizations in the private sectors. The precision of such frequency metrology could be further enhanced by state-of-the-art electron monochromators and aberration correctors^30^. In the presented work, we utilize a tabletop frequency comb source to synthesize optical frequencies, offering superior performance despite increased complexity. With recent advances in chip-scale frequency combs^50^ and microcomb generation within an electron microscope^46^, we foresee a compact and user-friendly solution for optical-driven EELS calibration, achieving a level of simplicity comparable to that of standard reference samples.

We anticipate our results would significantly improve the spectral resolution of electron spectroscopy, which already excels at the atomic spatial resolution. This improvement could further the spectroscopic capabilities of free electrons, particularly impactful for the study of low-energy excitations in solids, molecules, and nanostructures, including vibrational spectroscopy^24^, chemical shift analysis^23^, and quasiparticle search^51^. In addition, precision electron spectroscopy will be essential for the emerging field of free-electron quantum optics, which investigates quantum effects of free electrons and their coupling to other quantum systems, often manifested as electron energy states with minute energy differences^45,52,53^. Our work sets a milestone for establishing a precise definition and a new international standard for electron energy-change in the context of EELS. The IELS electron spectrum from the photonic chip-based device could calibrate the electron energy-loss spectrum of a specimen when two spectra are taken in succession. In-line calibration could also be implemented with a customized sample mount holding both the photonic chip and the specimen. Beyond calibration, the investigation lays the foundation for creating an ‘electron frequency comb’ when using photon energy sufficiently larger than the initial electron energy spread, thus enabling novel schemes of ultrahigh-precision frequency metrology with free electrons.

## Methods

### Photonic chip design, fabrication, and packaging

The Si_3_N_4_ photonic chip (wafer ID: D66_01_F10_C16) was fabricated by the photonic Damascene process^42,43^, with SiO_2_ bottom cladding and no top cladding. The microresonator has a ring radius of 20 μm. The nominal waveguide cross sections are 2 μm × 650 nm and 800 × 650 nm for the microresonator and the bus waveguide, respectively. The microresonator was characterized by a frequency-comb-assisted broadband optical spectroscopy with tunable diode lasers^54,55^. The measured total loss rates of the resonances used in the experiment are around  ~150 MHz, corresponding to a quality factor *Q* of  ~1.3 × 10^6^. The input and output fibers were prepared by splicing a short segment of ultrahigh numerical aperture (UNHA-7) fiber to a single mode fiber (SMF-28), and the photonic chip was packaged by attaching the fibers to the bus waveguide inverse taper with an ultraviolet-cured epoxy^44^. The packaged photonic chip was mounted on a custom-built TEM sample holder, and the measured fiber-to-fiber optical transmission is  ~18.6%.

### Transmission electron microscopy and electron energy-loss spectroscopy

The photonic chip was inserted into a TEM (JEOL JEM-2100PLUS) with a LaB_6_ thermionic electron gun. The electron beam was emitted by the gun in the continuous-beam mode and accelerated to 120 keV, allowing for phase matching with the microresonator optical modes. The beam current was in the range of 5–10 pA. The initial energy spread of the electron beam was 0.7–0.8 eV. During the experiment, the TEM was in low-magnification (×300 on the control panel) mode with the objective lens off. The electron beam was tightly focused close to the surface of the photonic chip with a spot size at the sample of  ~40 nm (FWHM) at a distance of 20−30 nm from the chip surface, and a convergence angle of  ~100 μrad (50 μm condenser aperture, spot size set with free-lens control of the condenser lenses). The electron spectra were measured with a post-column spectrometer (Gatan Imaging Filter GIF Quantum SE) with a 2048 × 2048 pixels CCD camera (US1000) and  ~5 mrad collection semi-angle. Each EELS spectrum was obtained by summing 5 frames of 2-s exposure each. The photonic chip was mounted on the tip of a custom-built sample holder with teflon fiber feedthroughs allowing bare optical fibers to enter the TEM column and be coupled to the photonic chip. During the experiments, the surface of the photonic chip was kept parallel to the TEM optical axis and to the electron beam optical axis. The electron energy-loss spectra were acquired in TEM mode with the electron beam focused tangent to the microresonator waveguide and parallel to its surface. The electron spectral broadening was maximized by fine-tuning the electron beam position and the photonic chip tilt.

### Optical setup

The CW laser was emitted by a tunable external cavity diode laser (Toptica) with a wavelength around 1550 nm and amplified by an erbium-doped fiber amplifier (Keopsys). The optical power sent to the input fiber was 50−80 mW. The optical polarization was controlled by a fiber polarization controller to excite the transverse magnetic mode family of the microresonator, and the laser frequency was tuned to specific resonant frequencies (shown in Fig. 4). A fraction of the output light was sent to a wavelength meter (HighFinesse) for coarse measurement of the optical frequency. The wavelength meter was calibrated by an internal neon lamp before each experimental session. The erbium-doped-fiber-laser-based optical frequency comb (Menlo Systems) was self-referenced and filtered by an optical bandpass filter. The frequency comb had a repetition rate *f*_rep_ = 251.6575 MHz and a carrier-envelope-offset frequency *f*_ceo_ = 20 MHz. The optical frequency comb and the output light were mixed at a fast photodiode (New Focus), with their spectral overlap monitored by an optical spectrum analyzer (Yokogawa) and their beatnotes measured by an electronic spectrum analyzer (Rohde, Schwarz). An optical phase-locked loop (OPLL) performed offset locking of the CW laser to one tooth of the optical frequency comb. Specifically, the lowest-frequency beatnote was brought to  ~10 MHz by tuning the laser piezo controller. The beatnote was then filtered, amplified, and compared with the reference, a 10 MHz local oscillator, using a double-balanced 12-bit digital phase detector (Menlo Systems). The digital phase detector has a large phase detection range and accounts for frequency fluctuation induced phase slips. The phase detector output, i.e., the error signal, was used as the input signal of a PID servo controller, of which the output signal was fed back to the laser current controller for actuation. The ambiguity of the beatnote frequency (± in Eq. (1)) was removed by observing the shift direction of the beatnote while adjusting the laser frequency when the OPLL was deactivated. An oscilloscope monitored both the error signal from the phase detector and the optical transmission measured by a photodiode.

### Data processing and analysis

Our calibration approach is analogous to the calibration of astronomical spectrometers using optical frequency combs^13,14,56^ (“astrocombs”). Just as astrocombs serve as precise frequency rulers for calibrating optical spectrometers, our method utilizes the energies of the IELS photon sidebands as accurate energy markers for calibrating electron spectrometers. Consequently, precise determination of the sideband energies is crucial for achieving highly accurate calibration results. The incident electrons, as well as the elastically scattered electrons, have a finite spectral width (0.7−0.8 eV) comparable to the photon energy (~0.8 eV). Therefore, the energy sidebands in the IELS electron spectrum are not fully separated and overlap each other. Furthermore, the ZLP and sidebands have a slightly asymmetric spectral shape. As a result, the electron spectral peak positions deviate from the regularly spaced energy grid defined by integer multiples of the photon energy. To mitigate this issue, we first smooth the raw data by convolution with a Gaussian pre-filter, and then use the Richardson-Lucy (RL) deconvolution to retrieve a comb-like spectrum with well-separated peaks^47^. The RL deconvolution uses the experimentally measured zero-loss peak (ZLP) spectrum as the deconvolution Kernel, after proper normalization and zero-padding. By separating the electron energy sidebands and eliminating the influence of a slightly asymmetric ZLP shape, the deconvolution enhances the precision of sideband position localization, providing accurate markers that are pivotal in astrocomb-like calibration techniques^13,14,56^. For electron spectrometer calibration, the peak positions of the deconvolved, comb-like electron spectrum are used as calibration markers. Pixel positions (with sub-pixel precision) of the calibration markers are assigned an energy of the corresponding integer multiples of the photon energy, which is determined from absolute optical frequency measurement. Pixels in between the calibration markers are assigned an energy from linear interpolation. As the result, the energy solution, i.e., the pixel-to-energy mapping, of the spectrometer is retrieved. The energy solution is then fitted with a third-order polynomial, with the slope of the linear component representing the calibrated average dispersion. The iterative RL deconvolution typically has an optimal number of iterations, at which point the deconvolution has to be terminated to prevent noise amplification^57^. For each electron spectrum, the deconvolution is tested for a range of parameter combinations with the Gaussian pre-filter width in 0.01–0.16 eV (standard deviation) and the number of iterations in 10–300. The optimal parameter set is determined by evaluating the calibration results, i.e., the calibrated average dispersion and energy solution, in three validation methods: (1) simulation-validation, where the deconvolution is performed on a simulated IELS electron spectrum with a known spectrometer energy solution as the ground truth; (2) self-validation, where a single experimental electron spectrum is deconvolved with different parameter combinations (this procedure is performed on 8 different spectra); (3) cross-validation, where a pair of experimental electron spectra generated with the same absolute optical frequency and recorded in rapid succession (less than 5 min time interval) are deconvolved and compared (this procedure is performed on 4 pairs of spectra). In consequence, the obtained range of optimal parameter combinations is 0.05–0.09 eV for the Gaussian pre-filter width and 60–100 for the number of iterations. The same range of parameter combinations is applied in the processing of all electron spectra. We use a range rather than a single parameter combination to ensure the robustness of the calibration method and to estimate the calibration uncertainty. The final calibration is the average of the calibrations obtained from all parameter combinations in the aforementioned range, and the maximal deviation from the average is taken as the uncertainty, which is 14.68 μeV/px in the average dispersion and 35.08 meV (0.7 pixel under nominal dispersion) pixel-wise root-mean-square uncertainty in the energy solution. These two uncertainty values are obtained from cross-validation and are the largest values from all three validation methods. Hence, the two uncertainty values represent a conservative estimate and do not exclude short-term jitter from instrument drift, instability, and lab environment.

## Supplementary information

Supplementary Information

Transparent Peer Review file

## Data Availability

The data generated in this study have been deposited in the Zenodo database (10.5281/zenodo.16600739).
