# Supplementary material for: Unifying frequency metrology across microwave, optical, and free-electron domains
Source: Nat Commun. 2025 Sep 24;16:8369. doi: 10.1038/s41467-025-62808-5 (PMC12460813; doi:10.1038/s41467-025-62808-5)
Supplement: Supplementary file 1 — Supplementary Information [file 41467_2025_62808_MOESM1_ESM.pdf]

Supplementary Information for:
Unifying frequency metrology across microwave, optical, and free-electron domains

Yujia Yang,[1,2,3,*] Paolo Cattaneo,[1,*] Arslan S. Raja,[1,2,3] Bruce Weaver,[1] Rui Ning Wang,[1,2,3]
Alexey Sapozhnik,[1] Fabrizio Carbone,[1] Thomas LaGrange,[1,†] and Tobias J. Kippenberg[1,2,3,‡]

[1] *Institute of Physics, Swiss Federal Institute of Technology Lausanne (EPFL), CH-1015 Lausanne, Switzerland*
[2] *Center for Quantum Science and Engineering, Swiss Federal Institute of Technology Lausanne (EPFL), CH-1015 Lausanne, Switzerland*
[3] *Institute of Electrical and Micro-Engineering, Swiss Federal Institute of Technology Lausanne (EPFL), CH-1015 Lausanne, Switzerland*

Contents

1. Theory on free-electron-light scattering 1

2. Photonic chip-based microresonator 3

3. Calibration uncertainty estimation 3
   3.1. Cross-validation 4
   3.2. Self-validation 4
   3.3. Simulation-validation 5
   3.4. Uncertainty estimation 5

4. Comparison with commonly used calibration techniques 6

5. Measurement of absolute optical frequency 7

References 7

Supplementary Note 1. Theory on free-electron-light scattering

Here, we recapitulate the theoretical description of the interaction between an electron wavepacket and a co-propagating continuous optical near-field, and demonstrate the formation of energy sidebands at integer multiples of the photon energy from the initial energy in the electron spectrum [1–5]. The evolution of the electron wavefunction $\psi(\mathbf{r}, t)$ is described by the time-dependent Schrödinger equation

$$i\hbar \frac{\partial \psi(\mathbf{r}, t)}{\partial t} = H\psi(\mathbf{r}, t), \tag{1}$$

with the Hamiltonian

$$H = \frac{1}{2m}\left(\mathbf{p} - q\mathbf{A}\right)^2 = \frac{\mathbf{p}^2}{2m} - \frac{q}{2m}(\mathbf{p}\cdot\mathbf{A} + \mathbf{A}\cdot\mathbf{p}) + \frac{q^2\mathbf{A}^2}{2m}, \tag{2}$$

where m and $q = -e$ are the mass and the charge of the electron, respectively, $\mathbf{p} = -i\hbar\nabla$ is the electron momentum operator, $\hbar$ is the reduced Planck constant, and $\mathbf{A}(\mathbf{r}, t)$ is the electromagnetic vector potential. The ponderomotive term proportional to $\mathbf{A}^2$ can be ignored since the electron momentum is much larger than $eA(\mathbf{r}, t)$, and the Hamiltonian reduces to $H = H_0 + H_1$, where $H_0 = \mathbf{p}^2/2m = -(\hbar^2/2m)\nabla^2$ is the free-electron Hamiltonian, and $H_1 = (e/2m)(\mathbf{p}\cdot\mathbf{A} + \mathbf{A}\cdot\mathbf{p})$ is the electron-light coupling term. Choosing the Coulomb gauge ($\nabla\cdot\mathbf{A} = 0$), the coupling term becomes $H_1 = (e/m)\mathbf{A}\cdot\mathbf{p} = (-ie\hbar/m)\mathbf{A}\cdot\nabla$. The electron wavefunction can be written as $\psi(\mathbf{r}, t) = \phi(\mathbf{r} - \mathbf{v}t, t)e^{i(\mathbf{k}_0\cdot\mathbf{r} - E_0 t/\hbar)}$, where $\mathbf{k}_0$ and E_0 are the central wavevector and energy of the electron, respectively, and $\phi(\mathbf{r}-\mathbf{v}t, t)$ is a slowly varying envelope propagating with the electron relativistic velocity $\mathbf{v}$. Substituting these elements in Eq. 1, the Schrödinger equation simplifies to an equation describing the evolution of the electron envelope, which reads as

$$\frac{\partial \phi}{\partial t} + \frac{\hbar}{m}\mathbf{k}_0\cdot\nabla\phi - \frac{i\hbar}{2m}\nabla^2\phi = -\frac{ie}{m}\mathbf{A}\cdot\mathbf{k}_0\phi - \frac{e}{m}\mathbf{A}\cdot\nabla\phi. \tag{3}$$

Since $\nabla^2\phi \ll \mathbf{k}_0 \cdot \nabla\phi$ and $|\nabla\phi| \ll |\mathbf{k}_0\phi|$ (slowly varying envelope), the third term on the left-hand side (dispersion term) and the second term on the right-hand side can be neglected and Eq. 3 reduces to

$$\frac{\partial\phi}{\partial t} + \mathbf{v}\cdot\nabla\phi = -\frac{ie}{\hbar}\mathbf{v}\cdot\mathbf{A}\phi, \tag{4}$$

where $\mathbf{v} = \hbar\mathbf{k}_0/m$ is the electron velocity. Switching to a reference frame moving with the electron velocity $\mathbf{r}' = \mathbf{r} - \mathbf{v}t$, Eq. 4 becomes

$$\frac{\partial\phi(\mathbf{r}', t)}{\partial t} = -\frac{ie}{\hbar}\mathbf{v}\cdot\mathbf{A}(\mathbf{r}' + \mathbf{v}t, t)\phi(\mathbf{r}', t), \tag{5}$$

which can be directly integrated as

$$\phi(\mathbf{r}', t) = \phi_0(\mathbf{r}')\exp\left[-\frac{ie\mathbf{v}}{\hbar}\cdot\int_{-\infty}^{t}\mathbf{A}(\mathbf{r}' + \mathbf{v}t', t')dt'\right], \tag{6}$$

where $\phi_0(\mathbf{r}') = \phi(\mathbf{r}', t \to -\infty)$ is the electron envelope before interacting with the optical field. The vector potential is related to the electric field by $\boldsymbol{\mathcal{E}} = -\partial\mathbf{A}/\partial t$, where the electric field can be expressed as $\boldsymbol{\mathcal{E}} = (1/2)\tilde{\boldsymbol{\mathcal{E}}}(\mathbf{r})e^{-i\omega t} + c.c.$ and with the complex field amplitude $\tilde{\boldsymbol{\mathcal{E}}}(\mathbf{r})$ only dependent on the position of monochromatic light. Therefore, the vector potential can be written as $\mathbf{A}(\mathbf{r}, t) = (1/2i\omega)\tilde{\boldsymbol{\mathcal{E}}}(\mathbf{r})e^{-i\omega t} + c.c.$. Without losses of generality, we can reduce the problem to one dimension by considering $\mathbf{v}$ along the z-axis and applying the substitution $t' = (z'' - z')/v$ in the integral, and Eq. 6 turns into

$$\phi(x, y, z', t) = \phi_0(x, y, z')\exp\left[-\frac{e}{2\hbar\omega}e^{i\frac{\omega}{v}z'}\int_{-\infty}^{z}dz''\tilde{\mathcal{E}}_z(x, y, z'')e^{-i\frac{\omega}{v}z''} + c.c.\right]. \tag{7}$$

We define the coupling constant $g(\mathbf{r})$ as the complex number

$$g(\mathbf{r}) \equiv \frac{e}{2\hbar\omega}\int_{-\infty}^{z}dz''\tilde{\mathcal{E}}_z(x, y, z'')e^{-i\frac{\omega}{v}z''}, \tag{8}$$

and $\mathcal{G}(\mathbf{r}) \equiv g(\mathbf{r})\exp(i\omega z'/v)$. Inserting these expressions in Eq. 7, we find the solution $\phi(x, y, z', t) = \phi_0(x, y, z')e^{-\mathcal{G}+\mathcal{G}^*} = \phi_0(x, y, z')e^{2i|\mathcal{G}|\sin(\arg(-\mathcal{G}))}$, which in the laboratory frame can be rewritten as

$$\phi(x, y, z - vt, t) = \phi_0(x, y, z - vt)e^{2i|g|\sin(\arg(-g) + \omega z/v - \omega t)}. \tag{9}$$

Eq. 9 shows that the optical near-field periodically modulates the longitudinal phase of the electron envelope in time (with the frequency of the optical field) and in space (with the wavevector change of the electron upon the absorption or emission of one photon with energy $\hbar\omega$). Finally, using the Jacobi-Anger identity and Bessel functions of the first kind J_N, the envelope function becomes $\phi(\mathbf{r} - \mathbf{v}t, t) = \phi_0(\mathbf{r} - \mathbf{v}t)\sum_{N=-\infty}^{+\infty} J_N(2|g|)e^{iN(\arg(-g) + \omega z/v - \omega t)}$, and the electron wavefunction after the interaction becomes

$$\psi(\mathbf{r}, t) = \phi_0(\mathbf{r} - \mathbf{v}t)\sum_{N=-\infty}^{+\infty} J_N(2|g|)e^{iN\arg(-g) + i\left[(k_0 + N\frac{\omega}{v})z - (E_0/\hbar + N\omega)t\right]}. \tag{10}$$

The final electron wavefunction of Eq. 10 is a coherent superposition of states with different energies and momenta. The exponential term shows that each Nth state, populated with probability $P_N = J_N(2|g|)^2$, has an energy and momentum at an integer multiple of the photon energy and the electron's momentum change upon the exchange of a photon from the initial electron's energy and momentum, respectively, namely $E_N = E_0 + N\hbar\omega$, and $\hbar k_N = \hbar k_0 + N\hbar\omega/v$.

A few comments on the approximations employed in the derivation are due. In the main text, we show a spectral broadening of > 50 eV on both the energy-gain and the energy-loss sides of the electron energy-loss spectrum, corresponding to the exchange of > 60 photons on each side. Despite the strong coupling ($g \sim 30$), the approximations used in the derivation of Supplementary Note 1 are still valid.

The slowly varying envelope approximation that allows to pass from Eq. 3 to Eq. 4 is valid when the momentum broadening of the wavepacket is small with respect to the central momentum. Similarly, the electron velocity can be considered constant during the interaction (nonrecoil approximation) when the momentum change after the interaction is negligible with respect to the central momentum. Upon the absorption/emission of a photon of energy $\hbar\omega$, the momentum change of the electron with velocity v is $\Delta p = \pm\hbar\omega/v$. Along the longitudinal axis, the relative change of

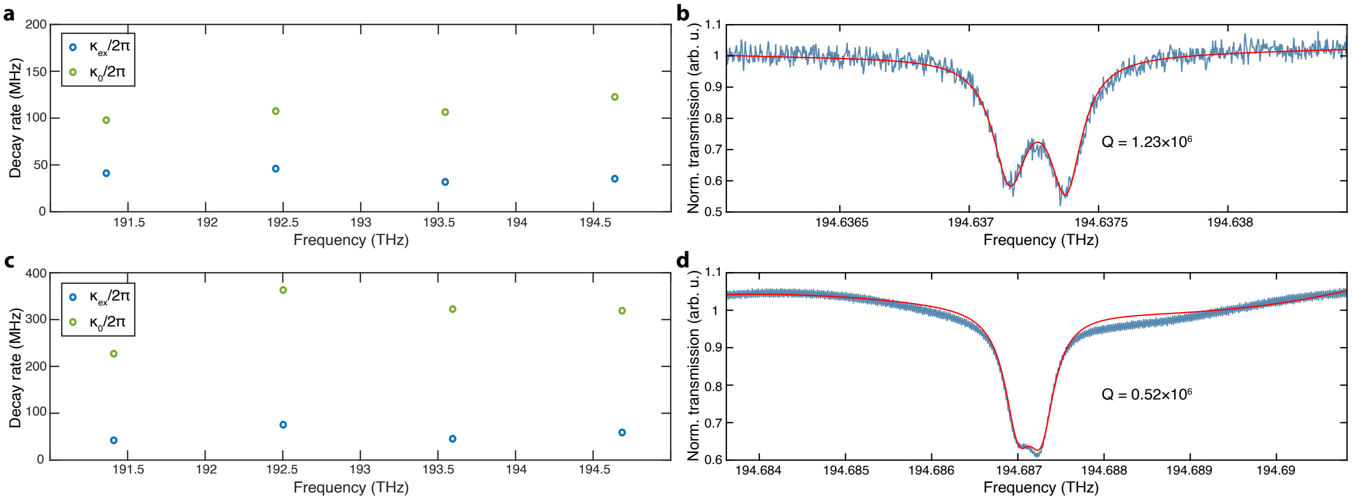

Supplementary Figure 1: Measured resonance linewidth and quality factor of the Si_3N_4 photonic chip-based microresonator. (a) Intrinsic loss rate $\kappa_0/2\pi$ and external coupling rate $\kappa_{\text{ex}}/2\pi$ before the experimental session. (b) Resonance near 194.64 THz with a quality factor of 1.23×10^6. (c) Intrinsic loss rate $\kappa_0/2\pi$ and external coupling rate $\kappa_{\text{ex}}/2\pi$ after the experimental session. (d) Resonance near 194.69 THz with a quality factor of 0.52×10^6.

momentum $\Delta p_{\|}/p = N\hbar\omega/\beta^2 mc^2\gamma$, where N is the number of photons absorbed/emitted by the electron, $\beta = v/c$, c the speed of light in vacuum, m is the electron mass, and γ the Lorentz factor. For $N \sim 60$, $\hbar\omega = 0.8$ eV, and 120 keV electrons, $\Delta p_{\|}/p \sim 2.2 \cdot 10^{-4} \ll 1$. Along the transverse direction (say the x-axis), we estimate the change of momentum as the effect of a classical electric force $e\mathcal{E}_x$ acting on the electron in the interaction length L. Assuming that the transverse component of the electric field has the same amplitude of the longitudinal ($\mathcal{E}_z = \mathcal{E}_x$), the deflection of the electron is $\theta = \Delta p_x/p = e\mathcal{E}_x L/vp = eE_z L/vp$. For a phase-matched light-electron interaction, the integral of Eq. 8 can be approximated as $g \sim e\mathcal{E}_z L/2\hbar\omega$, thus again $\Delta p_x/p = \theta \sim 2g\hbar\omega/\beta^2 mc^2\gamma \sim 0.2$ mrad $\ll 1$. A further proof of the validity of this approximations, is given by the excellent agreement between measurements and simulations performed according to the model presented here for a similar microresonator and for even higher g [6].

Supplementary Note 2. Photonic chip-based microresonator

The Si_3N_4 photonic chip-based microresonator has a radius of 20 µm and a free-spectral-range of $\sim$ 1.09 THz for the fundamental transverse magnetic (TM) mode. In the experiment, the frequency of the driving laser is tuned into one of the resonances to achieve intracavity field enhancement and strong electron phase modulation with a continuous-wave laser [6]. Supplementary Fig. 1a illustrates the measured intrinsic loss rate ($\kappa_0/2\pi$) and external coupling rate ($\kappa_{\text{ex}}/2\pi$) for 4 resonances, and Supplementary Fig. 1b shows an exemplary resonance near 194.64 THz with a quality factor $Q = 1.23 \times 10^6$. After the experimental session, the measured linewidth and resonance are shown in Supplementary Figs. 1c&d, respectively. We observe an increase in the intrinsic loss rate (i.e. a degradation of the quality factor) and a slight shift in the resonance frequency. We attribute these changes to the electron beam-induced hydrocarbon deposition on the surface of the photonic chip during the experiment.

Supplementary Note 3. Calibration uncertainty estimation

In our experiment, the zero-loss peak in the electron spectra has an energy spread of 0.7-0.8 eV, which is comparable to the photon energy or the spacing between energy sidebands of $\sim$ 0.8 eV. Therefore, the energy sidebands in the electron spectrum of inelastic electron-light scattering (IELS) overlap each other and cause the sideband peak positions to deviate from a regularly spaced energy grid defined by the photon energy. We use the Richardson-Lucy (RL) deconvolution to retrieve a comb-like electron spectrum with well-separated energy sidebands, and use them as an energy reference for electron spectrometer calibration. However, the deconvolved electron spectrum might still deviate from the ideal IELS spectrum with equidistant peaks and induce an error in the calibration results. Furthermore, additional errors could arise due to instrument drift, short-term instability, as well as lab environment including temperature fluctuations and stray electromagnetic fields. To estimate the uncertainty of our calibration technique, we investigate the errors in the calibration results, i.e. the calibrated average dispersion and energy solution, for

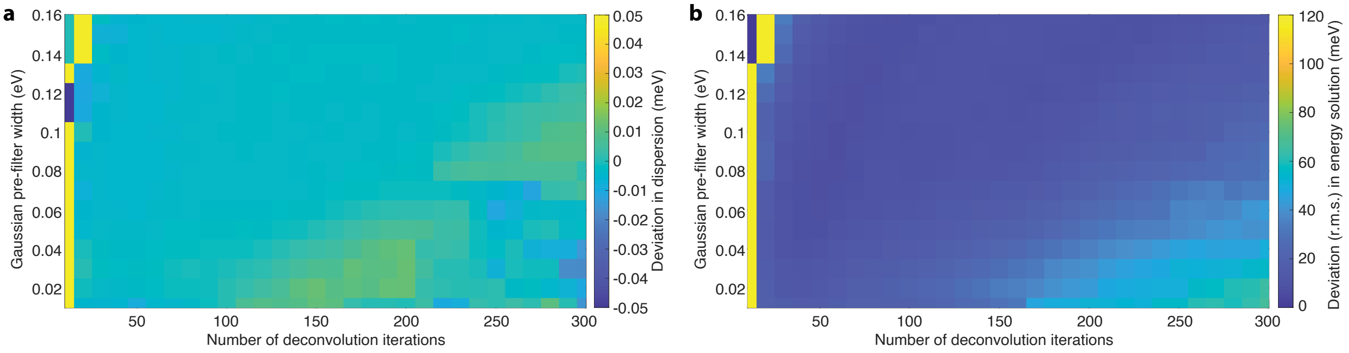

Supplementary Figure 2: Deviation in the calibration results with two exposures at the same absolute optical frequency (192 384 387 MHz) for a range of parameter combinations of the Gaussian pre-filter width and the number of deconvolution iterations. (a) Deviation in the calibrated average dispersion. (b) Root-mean-square (r.m.s.) deviation in the calibrated energy solution.

experimental or simulated IELS spectra. Specifically, we use three validation methods for uncertainty estimation: cross-validation, self-validation, and simulation-validation.

3.1. Cross-validation

For each absolute optical frequency, two experimental IELS spectra are recorded in rapid succession (less than 5 min time interval). Cross-validation performs independent spectrometer calibration based on the two exposures, and estimates uncertainty by evaluating the deviation between the two calibration results. This estimation assumes the two exposures should arrive at the same calibration results, namely, any short-term effects of instrument drift, instability, and lab environment are *included* in the estimated uncertainty. To evaluate the effect of deconvolution parameters, the calibrations are tested for a range of parameter combinations with the Gaussian pre-filter width in 0.01-0.16 eV and the number of deconvolution iterations in 10-300.

Supplementary Fig. 2 demonstrates the typical deviation in the calibration results for two exposures with the same absolute optical frequency (192 384 387 MHz) for a range of combinations of the Gaussian pre-filter width and the number of deconvolution iterations. In the ideal situation where the true energy solution of the spectrometer does not change between the two exposures, the deviation in both the calibrated average dispersion and the calibrated energy solution should be zero. In Supplementary Fig. 2, the deviation in the calibration results depends on the choice of both the Gaussian pre-filter width and the number of deconvolution iterations. The dependence on the number of iterations is characteristic for the RL deconvolution, which usually has an optimal number of iterations corresponding to the minimal error between the deconvolved data and the ground truth [7]. The deviation for a small number (e.g. < 30) of iterations comes from insufficient deconvolution, while the deviation for a large number (e.g. > 150) of iterations is due to noise amplification by the deconvolution algorithm. This optimal number of iterations increases with the Gaussian pre-filter width, since a larger number of deconvolution iterations is needed when the spectrum is first convolved with a wider Gaussian.

We choose a range of optimal parameter combinations of 0.05-0.09 eV for the Gaussian pre-filter width and 60-100 for the number of deconvolution iterations. This choice considers both a relatively small deviation in the calibration results for two consecutive exposures and a sufficiently large range of parameter combinations to ensure the robustness of the calibration method. We perform spectrometer calibration for 4 pairs of electron spectra acquired with an absolute optical frequency of 194 567 516 MHz, 193 474 819 MHz, 192 384 387 MHz, and 191 292 697 MHz using the same range of parameter combinations mentioned above. The deviations in the calibration results are averaged over this range, with the largest value among the 4 pairs corresponding to the estimated calibration uncertainty by the cross-validation method. This leads to an estimated uncertainty of 14.68 µeV/px in the average dispersion and 35.08 meV (pixel-wise r.m.s.) in the energy solution.

3.2. Self-validation

We also investigate the effect of deconvolution parameters by performing calibration using each electron spectrum with different combinations of the Gaussian pre-filter width and the number of deconvolution iterations. Self-validation method estimates the uncertainty by evaluating the sensitivity of the calibration results to the choice of deconvolution parameters.

Supplementary Fig. 3 shows the typical calibration results with an individual electron spectrum acquired at

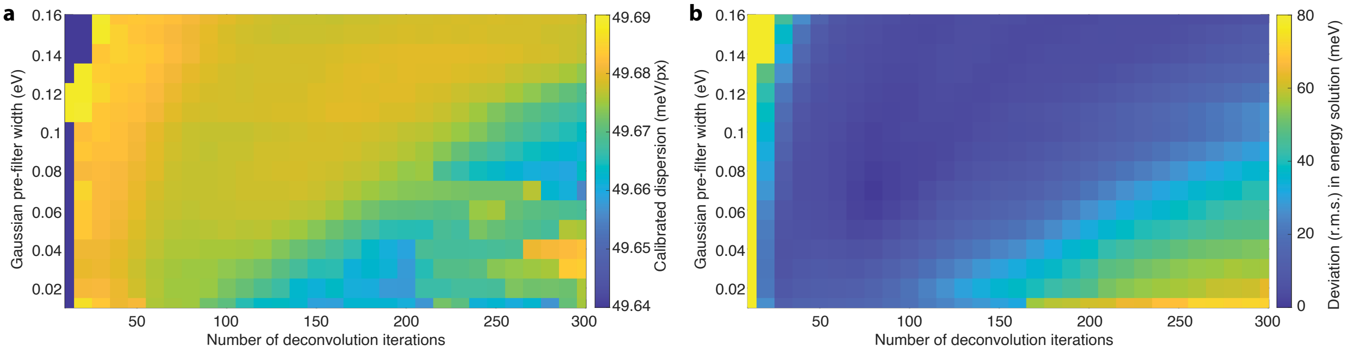

Supplementary Figure 3: Calibration results with an electron spectrum acquired at an absolute optical frequency of 192 384 387 MHz **for a range of parameter combinations of the Gaussian pre-filter width and the number of deconvolution iterations.** (a) The calibrated average dispersion. (b) Root-mean-square (r.m.s.) deviation in the calibrated energy solution from the calibration performed with 0.07 eV Gaussian pre-filter width and 80 deconvolution iterations.

an absolute optical frequency of 192 384 387 MHz for a range of parameter combinations of the Gaussian pre-filter width and the number of deconvolution iterations. An accurate and robust calibration method should have minimal dependence on the deconvolution process when the parameters are chosen properly. For both the calibrated dispersion and the r.m.s. deviation in the calibrated energy solution (from the calibration performed with 0.07 eV Gaussian pre-filter width and 80 deconvolution iterations), the range of proper parameter combinations is similar to the one shown in the cross-validation method - there is an intermediate range of optimal number of iterations and it shifts to larger numbers with an increasing Gaussian pre-filter width.

We choose the same range of parameter combinations as before, i.e. 0.05-0.09 eV for the Gaussian pre-filter width and 60-100 for the number of deconvolution iterations. We perform spectrometer calibration with each of the 8 electron spectra for all parameter combinations within that range. The calibration results are averaged over the parameter range, and the maximal deviation from the mean value is considered as the estimated uncertainty, which is 2.8 µeV/px in the average dispersion and 3.78 meV (pixel-wise r.m.s.) in the energy solution.

3.3. Simulation-validation

We further investigate the calibration uncertainty by performing the calibration with a simulated IELS electron spectrum. For the simulated spectrum, we use a perfectly linear energy solution with a 50 meV/px dispersion as the known ground truth, i.e. the true energy solution of the spectrometer. The simulated IELS spectrum considers a Gaussian distribution of the electron beam position, partial blocking of the electron beam by the photonic chip, and an exponential decay of the optical near-field away from the chip surface. Gaussian noise is also added to the simulated spectrum and has a standard deviation similar to the electron-beam shot noise estimated from experimental conditions.

Supplementary Fig. 4 shows the calibration results with the simulated IELS spectrum for a range of parameter combinations of the Gaussian pre-filter width and the number of deconvolution iterations. A perfectly accurate calibration method should be able to retrieve the known ground truth, resulting in a calibrated average dispersion of 50 meV/px and a zero r.m.s. deviation from the true energy solution. We again choose the same range of parameter combinations, i.e. 0.05-0.09 eV for the Gaussian pre-filter width and 60-100 for the number of deconvolution iterations, and evaluate the mean deviation of the calibration results from the known ground truth. The simulation-validation method uses this mean deviation as an estimate of the calibration uncertainty, which is 2.29 µeV/px in the average dispersion and 5.85 meV (pixel-wise r.m.s.) in the energy solution.

3.4. Uncertainty estimation

We consider all the uncertainty values estimated by the three methods discussed above, and take the largest value as a conservative estimate of the calibration uncertainty: 14.68 µeV/px in the average dispersion and 35.08 meV (pixel-wise r.m.s.) in the energy solution. Both of these uncertainties are obtained from the cross-validation method that compares the calibration results based on two independent exposures recorded in rapid succession. Therefore, the estimated uncertainty *includes* short-term effects of instrument drift, instability, and lab environment.

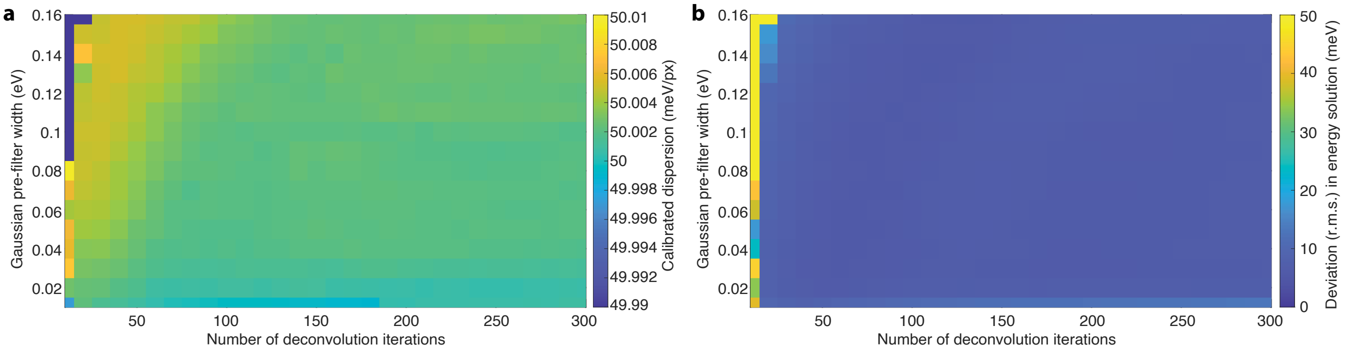

Supplementary Figure 4: Calibration results with a simulated electron spectrum for a range of parameter combinations of the Gaussian pre-filter width and the number of deconvolution iterations. (a) The calibrated average dispersion. (b) Root-mean-square (r.m.s.) deviation in the calibrated energy solution from the known ground truth.

Supplementary Note 4. Comparison with commonly used calibration techniques

Here, we compare our calibration technique with those commonly used for calibrating EELS spectrometers. Conventional approaches often overlook two important sources of error in the dispersion calibration. The first is the sensitivity of the EELS system to time-dependent ambient conditions in the microscope room, such as temperature, humidity, stray electromagnetic fields, even from the position of ferromagnetic elements close to the system, to the hysteresis of the magnetic lenses and from electronic instabilities. The second is the nonlinearity and local irregularity of the dispersion (in terms of eV/pixel) induced by intrinsic nonlinearity and aberrations of the magnetic prism and other electron-optical elements in the electron spectrometer as well as imperfections of the electron detector. Despite the remarkable development of aberration correctors, high-order aberrations still cause appreciable changes in the dispersion in different regions of the EELS spectrum.

The standard techniques for calibrating electron spectrometers can be divided into two categories based on the energy reference used. The first category utilizes as energy reference the positions of core-loss edges of standard samples [8, 9]. The calibration consists of using the white line of two core-loss edges with known energy values in a single spectrum and extracting the dispersion by simply dividing the energy difference by the pixel distance between the peaks of the edges' white lines. This is the most straightforward and commonly used technique. The most used edges include the graphite π^* peak, the Ni L_3 edge, and the O K edge in NiO [8]. This calibration has several limitations. The first limitation is the shift of the edge position that depends on the exact chemical environment of the sample used (oxidation state, bonding geometry, ligands type), known as chemical shift. Despite the use of high-quality commercially prepared standards, resulting chemical shifts can be in the eV range [10, 11], and edge positions in different publications can differ by several eV [12]. This uncertainty on the absolute edge position propagates to the EELS calibration, whose energy solution cannot be better than $\sim$ eV. Furthermore, having only two energy markers in the calibration, the technique is unable to detect changes in the dispersion across the spectrum, and the assumption of a linear energy-to-pixel mapping is intrinsically false and introduces a further source of error that can reach several eV across the spectrum [13]. We estimate the uncertainty in the dispersion, considering that the error in energy is typically integrated over a range of pixels, spanning from a few hundred to several thousand. As a result, the uncertainty in the dispersion will be approximately three orders of magnitude smaller than the corresponding uncertainty in the absolute energy. Consequently, the uncertainty in the dispersion when using core-loss edges is never better than a few meV/px.

The second calibration approach implements an energy reference from the voltage applied to the drift tube of the spectrometer [13–17]. The drift tube voltage is used to shift the energy range of the spectrum without changing the illumination conditions or introducing lens hysteresis effects. This is accomplished by applying a precise voltage to the drift tube that temporarily accelerates the beam as it passes through the prism, resulting in a net downward deflection of the dispersed electron beam. This calibration method applies incremented drift tube voltage steps and records a spectrum for each voltage step, from which it is possible to map the detector pixel to the energy. The advantage of this approach is that, with an arbitrary number of energy markers, it is possible to detect nonlinearities and perform a more accurate pixel-to-energy mapping. However, such calibration requires multiple acquisitions and makes it more susceptible to the instabilities of the drift tube voltage and the energy of the primary electrons. Furthermore, the drift tube itself requires calibration, and its accuracy also depends on the accuracy of this calibration. For example, in ref. [17] such calibration is carried out using the Ni L_3 edge of NiO and the π^* peak of graphite and introducing the same sources of error discussed above. These factors contribute to cumulative errors and limit the accuracy of

this calibration approach to a few hundred meV. The corresponding uncertainty on the dispersion is of hundreds of µeV/px.

With respect to the conventional techniques, the approach in our work has several key advantages. (1) The energy reference we use (the photon energy) is synthesized with extreme accuracy, i.e. in the neV range that is only limited by the frequency noise of the beatnote of the continuous-wave laser and the optical frequency comb. (2) A single EELS acquisition is sufficient for calibrating the entire spectral range covered by the energy sidebands, making the calibration more robust against environmental and electronic instabilities. (3) Having more than a hundred energy markers in a single spectrum, our technique can measure the nonlinearities and local irregularities of the dispersion across the energy range. (4) The performance of the calibration is verified by a precise energy standard with a known absolute energy; this verification step has never been achieved or proposed in previous demonstrations. These advantages contribute to the unprecedented uncertainty of 35.08 meV and of 14.68 µeV/px on the energy solution and on the dispersion, respectively.

Supplementary Table I summarizes the differences between our work and other techniques.

Energy reference	Optical frequency	Core-loss edge	Drift tube voltage
**References**	Our work	Refs. [8, 9]	Refs. [13–17]
**Energy solution uncertainty**	35.08 meV	$\gtrsim$ 1 eV	$\gtrsim$ 100 meV
**Dispersion uncertainty**	14.68 µeV/px	$\gtrsim$ 1 meV/px	$\gtrsim$ 100 µeV/px
**Number of acquisitions**	Single	Single	Multiple
**Measure irregularities and nonlinearities?**	Yes	No	Yes
**Verification by a precise absolute energy?**	Yes	No	No
**Main source of error**	Deconvolution method, short-term drift of instrument and lab environment	Chemical shifts, short-term drift of instrument and lab environment	Calibration of the drift tube, instabilities of its voltage and the primary beam energy, short-term drift of instrument and lab environment

Supplementary Table I: Comparison between our work and other calibration methods for EELS spectrometers.

Supplementary Note 5. Measurement of absolute optical frequency

In the main text, we show a proof-of-concept demonstration of ultrahigh-precision electron spectroscopy by measuring the absolute optical frequency from the electron spectra before and after calibrating the EELS spectrometer with a reference IELS spectrum. Before spectrometer calibration, the uncertainty of this type of measurement is subject to the accuracy of the nominal dispersion. Calibration of the spectrometer reveals a systematic error of $\Delta E \approx 300\,\mu\text{eV/px}$ in the nominal dispersion, and the measurement uncertainty is estimated as $\Delta E \cdot E_{\text{ph}}/E_{\text{disp\_norm}}$ with the photon energy E_{ph} and the nominal dispersion $E_{\text{disp\_norm}}$. After spectrometer calibration, the uncertainty of the optical frequency measurement is considered as $\delta E \cdot E_{\text{ph}}/E_{\text{disp}}$ with the estimated calibration uncertainty $\delta E = 14.68\,\mu\text{eV/px}$ and the calibrated dispersion E_{disp}.

Supplementary References

* These authors contributed equally.
† thomas.lagrange@epfl.ch
‡ tobias.kippenberg@epfl.ch

[1] S. T. Park, M. Lin, and A. H. Zewail, New Journal of Physics **12**, 123028 (2010).
[2] F. J. García de Abajo, A. Asenjo-Garcia, and M. Kociak, Nano Letters **10**, 1859 (2010).
[3] S. T. Park and A. H. Zewail, The Journal of Physical Chemistry A **116**, 11128 (2012).
[4] F. J. García de Abajo, B. Barwick, and F. Carbone, Physical Review B **94**, 041404 (2016).
[5] G. M. Vanacore, I. Madan, G. Berruto, K. Wang, E. Pomarico, R. J. Lamb, D. McGrouther, I. Kaminer, B. Barwick, F. J. García de Abajo, and F. Carbone, Nature Communications **9**, 2694 (2018).

[6] J.-W. Henke, A. S. Raja, A. Feist, G. Huang, G. Arend, Y. Yang, F. J. Kappert, R. N. Wang, M. Möller, J. Pan, J. Liu, O. Kfir, C. Ropers, and T. J. Kippenberg, Nature **600**, 653 (2021).

[7] D. S. C. Biggs and M. Andrews, Applied Optics **36**, 1766 (1997).

[8] R. Egerton, *Electron Energy-Loss Spectroscopy in the Electron Microscope* (Springer US, Boston, MA, 2011).

[9] R. F. Egerton and S. C. Cheng, Ultramicroscopy **55**, 43 (1994).

[10] T. L. Daulton and B. J. Little, Ultramicroscopy **106**, 561 (2006).

[11] H. Tan, J. Verbeeck, A. Abakumov, and G. Van Tendeloo, Ultramicroscopy **116**, 24 (2012).

[12] H. K. Schmid and W. Mader, Micron Proceedings of the International Workshop on Enhanced Data Generated with Electrons (EDGE), **37**, 426 (2006).

[13] RWH. Webster, AJ. Craven, B. Schaffer, S. McFadzean, I. MacLaren, and DA. MacLaren, Ultramicroscopy **217**, 113069 (2020).

[14] P. E. Batson, S. J. Pennycook, and L. G. P. Jones, Ultramicroscopy **6**, 287 (1981).

[15] P. L. Potapov and D. Schryvers, Ultramicroscopy **99**, 73 (2004).

[16] O. L. Krivanek, T. C. Lovejoy, N. Dellby, and R. Carpenter, Microscopy **62**, 3 (2013).

[17] G. Kothleitner, W. Grogger, M. Dienstleder, and F. Hofer, Microscopy and Microanalysis **20**, 678 (2014).
